# Rapid inversion of singleton distractor representations underlies learned attentional suppression

**DOI:** 10.1101/2025.10.08.680699

**Published:** 2025-12-22

**Authors:** Ziyao Zhang, Jarrod A. Lewis-Peacock

**Affiliations:** Department of Psychology, The University of Texas at Austin

## Abstract

In visually complex and dynamically changing environments, humans must often filter out salient but task-irrelevant stimuli. Prior work shows that with repeated exposure to search arrays containing color singleton distractors, individuals can learn to divert attention away from these salient items. However, the neural mechanisms supporting such attentional suppression remain unclear. The present study examined the temporal trajectories of singleton distractor representations during visual search to address this gap. Using multivariate pattern analyses of EEG data (N = 40), we identified two clusters of decodable singleton distractor representations: an early cluster from 100–200 ms and a later cluster from 200–400 ms. Temporal generalization analyses showed that the later representations were transformed versions of the early ones. Importantly, stronger late, but not early representations, predicted faster search responses, suggesting that the later signals support distractor suppression. We hypothesized that this transformation facilitates suppressing singleton distractors in the spatial priority map. Comparing decoding evidence across locations revealed that target locations were consistently enhanced relative to non-singleton distractors, whereas singleton distractor locations were suppressed. Moreover, comparing the neural coding of locations revealed that the spatial organization in the singleton distractor neural space was inverted relative to that in the target neural space. Together, these findings reveal a rapid representational transformation underlying salient distractor suppression at the onset of visual search. This rapid inversion of singleton distractor signals was likely driven by top-down control mechanisms that transform bottom-up saliency signals, producing an inverted arrangement of target and distractor information within a shared neural space.

## Introduction

The ability to filter out distracting information is crucial for goal-directed behavior, particularly in today’s digitally saturated environment. Stimuli such as website pop-ups inherently capture attention, raising the question of whether salient stimuli can be effectively suppressed within the visual system to support goal-directed actions. Recent insights into this debate stem from research on suppressing color singleton distractors (e.g., a red item among green items) in goal-oriented search tasks (Bacon & Egeth, 1994; Gaspelin & Luck, 2018b, 2018a; Luck et al., 2021). Color singleton distractors readily capture attention and impede visual searches (Jonides & Yantis, 1988; Theeuwes, 2010). However, emerging evidence suggests that proactive inhibitory processes can mitigate attentional capture by color singleton distractors. Repetitive training on search arrays containing color singleton distractors led participants to be faster at detecting a shape target when a color singleton distractor was present, compared to when it was absent (Chang & Egeth, 2019; Gaspelin et al., 2015, 2017; Vatterott & Vecera, 2012). This behavioral benefit in singleton-present trials provides initial evidence for suppression. However, it is important to note that mechanisms other than suppression could also produce similar reaction time benefits. These include ignoring singleton distractors in a serial search mode (Theeuwes, 2023), down-weighting the distractor dimension when target saliency is high (Liesefeld & Müller, 2019; 2021), and the rapid rejection of singleton distractors (Liesefeld et al., 2021; 2025).

Eye-tracking data showed that initial saccades were less likely to land on singleton distractors than on non-singleton distractors (Gaspelin et al., 2017; Stilwell et al., 2023). However, measures of overt attention cannot rule out the possibility of rapid covert capture by singleton distractors or the involvement of fast, reactive distractor handling mechanisms (e.g., Klink et al., 2023). Additionally, an ERP component, Pd, was identified in tasks with observed behavioral attentional suppression, suggesting that the suppression of color singleton distractors might be associated with early inhibitory processes occurring around 100–300 ms into the search (Cosman et al., 2018; Gaspelin et al., 2023; Sawaki et al., 2012; Sawaki & Luck, 2010). However, whether Pd necessarily reflects active salience suppression remains debated. Alternative interpretations propose that Pd may instead index the upweighting of nontargets rather than the suppression of distractors (Kerzel & Burra, 2020; Kerzel & Huynh Cong, 2023), or reflect sensory imbalances between visual hemifields (Oxner et al., 2025; for a recent review of Pd, see Gaspelin et al., 2023).

These findings, including behavioral benefits in singleton-present trials, oculomotor suppression of singleton distractors, and the presence of the Pd, have motivated the proposal of the signal suppression hypothesis (Gaspelin & Luck, 2018b; Sawaki et al., 2012; Sawaki & Luck, 2010). This framework posits that physically salient stimuli automatically generate bottom-up priority signals that can capture attention, but top-down mechanisms can suppress these signals before attention is deployed. According to this account, suppression acts by downweighting the feature dimension of singleton distractors prior to the initial shift of spatial attention (Gaspelin et al., 2018b; Gaspelin et al., 2025). In contrast, the rapid disengagement hypothesis proposes that suppression occurs reactively, after attention has been captured by the singleton distractor (Theeuwes, 2010; Theeuwes et al., 2000; see also Liesefeld et al., 2025 for a related account). Recent computational work has partially reconciled these views by modeling both suppression and capture within a single framework using data from forced-response paradigms (Zhang et al., 2025). In this model, suppression is achieved by preventing an early distractor priority signal from triggering an actual saccade toward the distractor. Model fits suggest that in cases of oculomotor suppression, a rapid target priority signal overrides the earlier distractor signal. In contrast, oculomotor capture emerges when the target-related signal arrives too late, allowing the distractor signal to drive early saccades. These results imply that the competing accounts of suppression may not reflect qualitatively distinct mechanisms; rather, they may lie on a continuum, primarily governed by the relative timing of target and distractor-related priority signals (Zhang et al., 2025).

Despite significant progress in the attentional capture debate, the neural mechanisms underlying attentional suppression remain unclear. Neural recordings and imaging studies have suggested two potential mechanisms for attention suppression. First, suppression is likely supported by attenuation of distractor-related signals. Singleton distractor signals may be inhibited during the initial stage of sensory processing due to adaptation (Turatto et al., 2018; Vatterott & Vecera, 2012; Won et al., 2019). Consistent with the adaptation account, fMRI studies demonstrated that repeated singleton distractors were suppressed starting in V1, as well as in intraparietal cortex (Adam & Serences, 2021; Won et al., 2020), regions that are important for computing bottom-up salience (Zhang et al., 2012; Li, 2002; Poltoratski et al., 2017; Sprague et al., 2018). These findings suggest that singleton distractor suppression may be driven by reduced feedforward activity (Adam & Serences, 2021; Won et al., 2020). Similarly, neural recordings in non-human primates (NHPs) have shown that singleton distractor signals are inhibited in regions responsible for computing spatial priorities (Cosman et al., 2018; Ipata et al., 2006). Recordings of neuronal activity in the lateral intraparietal area (LIP) and frontal eye fields (FEF) in NHPs have shown that color singleton distractors elicited weaker activity compared to non-singleton distractors when appearing in the receptive field of neurons. This suggests that suppression of singleton distractors occurs in regions implicated in spatial priority computations.

In contrast to signal attenuation, recent evidence suggests that attentional suppression may rely on mechanisms of signal transformation. A recent study reported that singleton distractors elicited enhanced neural activity early in the search, followed by later suppression relative to non-singleton distractors in area V4 of NHPs (Klink et al., 2023). Further examination of temporal response profiles in FEF and LIP revealed three equally sized neuronal subpopulations exhibiting sustained enhancement, sustained suppression, or early enhancement followed by later suppression to singleton distractors compared to non-singleton distractors (Sapountzis et al., 2025). This heterogeneity strongly suggests that signal attenuation cannot fully account for how singleton distractors are processed. Moreover, decoding analyses have shown that target and singleton distractor representations coexist in a partially shared neural format, indicating that both representations remain behaviorally relevant and, critically, that singleton distractor representations are not attenuated through training (Sapountzis et al., 2025). This raises important open questions about how these distractor representations contribute to behavioral suppression and why they do not compete for attentional allocation. It is likely that distractor representations are transformed into a format that does not interfere with, and may even facilitate, target guidance. Such a transformation mechanism offers a new lens for interpreting existing theories, including both the signal suppression and rapid disengagement accounts, which posit that the initial priority signal generated by salient singleton distractors must ultimately become a suppression signal in the priority map. Suppression could occur because the feedforward signals from singleton locations are attenuated; alternatively, the distractor signals may be recoded into a format that inherently produces suppression within the priority map. Here, we provide evidence supporting such a transformation.

The mechanisms underlying the potential signal transformation of distractors remain both puzzling and intriguing. To examine this, we used temporal generalization analyses of EEG data to test potential representational shifts during a distractor-filled visual search process. Temporal generalization is particularly useful for disentangling signal attenuation from signal transformation as potential mechanisms of attentional suppression. The signal attenuation account suggests that distractor signals gradually weaken during the search, but should maintain neural representations that can generalize across time. Conversely, the signal transformation mechanism posits that singleton distractor representations dynamically change during the search, leading to orthogonal or even negatively correlated neural representations across time. To test these competing hypotheses, we utilized multivariate pattern analysis (MVPA) on EEG data to assess changes in singleton distractor representations during a visual search task.

## Materials and Methods

We reanalyzed data shared from Stilwell et al., 2022. Two groups of 20 participants were recruited for experiment 1 and experiment 2, respectively. In each trial, participants searched for a predefined target item (circle or diamond, counterbalanced between participants) in a search array. In the search array, the inner ring contained a target shape and distractors that were in different shapes. On 75% of trials, a color singleton distractor that had a distinct color from other items was presented in the inner ring. The color singleton distractor was never the target, and participants were instructed to use the shape feature to locate the target item. The specific color of the color singleton distractor (red or green) was fixed for each participant but counterbalanced across participants. The outer ring contained non-target shapes only to boost the relative salience of the singleton distractor. Overall, participants completed 1296 singleton-present trials and 432 singleton-absent trials. Search arrays in experiment 1 had an inner ring with four items (effective set size 4), while in experiment 2, the inner ring had eight items (effective set size 8). EEG signals were collected and preprocessed following the procedures outlined in Stilwell et al., 2022.

### Decoding of target and singleton distractor locations

Location decoding was conducted separately for singleton and singleton-absent trials. For singleton-absent trials, we used support vector machine (SVM) to classify the target item’s location based on the spatial distribution of the EEG signal across 17 posterior electrodes (Pz, P3, P5, P7, P9, PO7, PO3, O1, POz, Oz, P4, P6, P8, P10, PO4, PO8, O2). We implemented this model using the *svc* function from the *sklearn* package in Python. EEG data were downsampled to 100 Hz, and decoding was performed for each time point (10 ms) ranging from −200 ms to 800 ms relative to the onset of the search array. For each time point, the dataset was divided into 3 folds. An equal number of trials from each location class were randomly selected for each fold without replacement to ensure unbiased training. Two folds were used as the training set, and the remaining fold served as the testing set. This procedure was repeated for the 3 folds, with each one serving as the testing set in an iteration. The entire process was repeated 100 times (Wolff et al., 2020), and decoding accuracies were averaged across 100 iterations. Decoding of target and singleton distractor locations in singleton-present trials followed the same procedure as decoding target locations in singleton-absent trials, except for adjusting the training set to match the number of trials in singleton-absent trials. This adjustment aimed to ensure equal training data for decoders in both singleton and singleton-absent trials. This should prevent biases in decoding performance comparisons between singleton and singleton-absent trials resulting from uneven training sets.

### Linear models linking decoding evidence to reaction time

To link target location representations to reaction time, decoding evidence for the correct target location in each trial was obtained from the *decision_function* in *sklearn*, which reflects the model’s confidence (distance to the decision hyperplane). Higher values indicate stronger neural representations of the target location. Decoding evidence was averaged within the early (100 – 200 ms) and later (200 – 400 ms) time windows. Both the averaged decoding values and reaction times were z-scored within participants before fitting the linear models. Linear mixed effects models were then fitted using the *lmer* function in *R*, with early and late decoding evidence as predictors and reaction time as the outcome variable. To link singleton distractor representations to reaction time, we applied the same analysis pipeline, except that decoding evidence was extracted for singleton distractor locations rather than target locations.

### Latency estimation

To estimate the onset latency of above-chance decoding, we applied a jackknife-based bootstrapping procedure (Miller et al., 1998; Ulrich & Miller, 2001). We generated N bootstrapped subsamples (N = 20 in both experiments), each created by leaving out one participant. For each subsample, we performed a one-sample t-test comparing decoding accuracy to chance. The latency for that subsample was defined as the onset of the earliest cluster of significant time points. This procedure yielded N latency estimates. These estimates were then used for statistical comparisons.

### Temporal generalization analysis

Temporal generalization analyses were conducted for target locations in singleton-absent trials and singleton locations in singleton-present trials. The same decoding procedures were used, with the only difference being that trained decoders for each time point were used to predict location labels for data at all time points.

### Cross-condition generalization

Cross-condition generalization was performed between target locations in singleton-absent trials and singleton distractor locations in singleton-present trials. SVM decoders were trained exclusively on data from singleton-absent trials with labels indicating target locations. These trained decoders were then used to predict singleton distractor locations in singleton-present trials. Decoding accuracies were computed based on the probability of the decoders’ predictions matching the actual singleton distractor location labels.

### Activation scores

To compute activation scores for each location in singleton-present trials, decoders were first trained on target locations in singleton-absent trials. These trained decoders were subsequently used to predict location labels in singleton-present trials. Instead of generating prediction labels, prediction probabilities were produced to indicate the likelihood of a given location being the true label based on the neural patterns. Prediction probabilities for target locations, singleton locations, and non-singleton distractor locations were averaged across trials for each subject. For plotting the spatial priority map, the averaged prediction probabilities of non-singleton distractor locations (two in experiment 1, six in experiment 2) were used as the baseline activation. The activation for target and singleton distractor locations was computed as the difference between the prediction probabilities of these locations and the baseline.

### Correlations between target and singleton representations

Raw EEG data of singleton-absent trials (200 – 400 ms) with the same target location were averaged to create the mean target representations for each potential target location (four in experiment 1 and eight in experiment 2). Likewise, singleton-present trials with the same singleton distractor location were averaged (200 – 400 ms) to generate singleton distractor representations for all potential singleton distractor locations (four in experiment 1 and eight in experiment 2). Pearson correlations in multi-channel EEG activities between target and singleton representations were computed for each potential location and then averaged. Positive correlations indicate similarity between the location representations in the target space and location representations in the singleton distractor space, while negative correlations suggest inverted representations.

Principal Component Analysis (PCA) was utilized to visualize the relation between the target representational space and the singleton distractor representational space. For this purpose, we identified the first two principal components (PCs) of the target representational space for one example participant (99% variance explained). Both target and singleton distractor representations were projected into the space defined by these two PCs. It should be noted that PCA was used to visualize the relationship between the target and singleton distractor representation only. The formal correlation analysis was performed based on the raw signal space.

### Statistics

We conducted one-sample (one-sided) t-tests to compare decoding accuracies against the theoretical chance level, as below-chance decoding is not meaningful for this analysis. All other comparisons were performed using two-sided t-tests. One-sample t-tests were also used to compare temporal generalizability scores and cross-condition generalizability scores against the theoretical chance level. In both cases, above-chance scores indicated positive generalizability, whereas below-chance scores indicated negative generalizability. Paired t-tests were used to compare prediction probabilities for target locations, singleton distractor locations, and non-singleton distractor locations. Finally, one-sample t-tests were conducted to compare neural correlations between target and singleton distractor representations against zero, with positive values indicating positive correlations and negative values indicating negative correlations. All analyses were performed in Python using standard statistical packages. Bayes factors for the alternative hypothesis (*BF10*) were reported, with values between 1 and 3 indicating anecdotal evidence, values between 3 and 10 indicating moderate evidence, and values greater than 10 indicating strong evidence. To account for multiple comparisons, Sidak correction was applied, and adjusted p-values were reported.

## Results

Participants searched for a predefined target item (circle or diamond, counterbalanced between participants) in a search array. In the search array, the inner ring contained a target shape and distractors that were in different shapes. On 75% of trials, a color singleton distractor that had a distinct color from other items was presented in the inner ring. The color singleton distractor was never the target, and participants were instructed to use the shape feature to locate the target item. The outer ring contained non-target shapes only to boost the relative salience of the singleton distractor.

### Singleton distractor representations showed distinct temporal patterns compared to target representations.

**Fig. 1.**
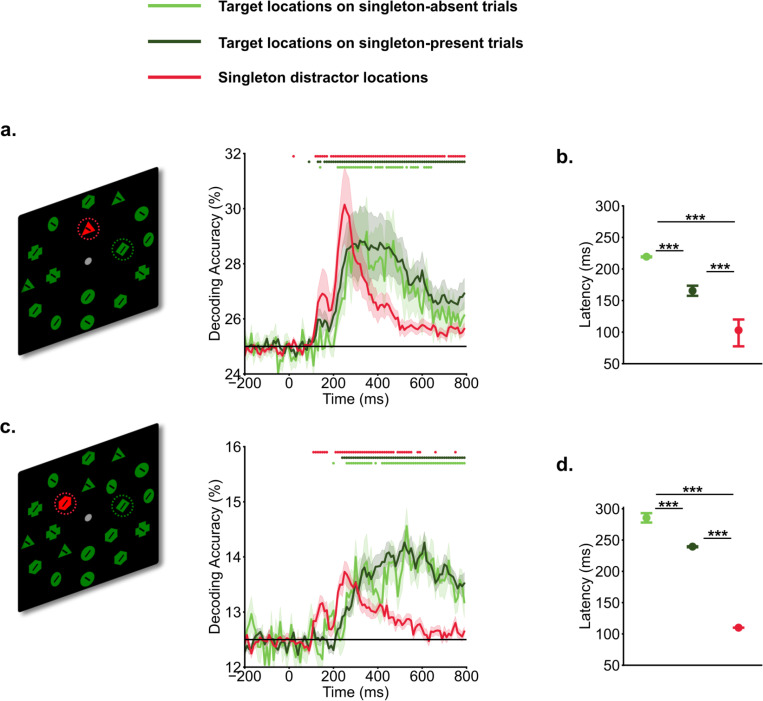
Temporal profiles of target and singleton distractor representations. Participants searched for a specific target shape (e.g., a green diamond) within the inner ring of a search array, and made a speeded button press to indicate the direction of the line within the target shape (left vs. right). Time 0 ms represents the onset of search arrays. In experiment 1 (a. set size 4) and experiment 2 (b. set size 8). a&c). decoding of singleton distractors showed an earlier initial peak of decoding evidence from 100 ms to 200 ms, followed by a later peak from 200 ms to 400 ms. Target decoding showed a gradual increase in evidence The horizontal black line indicates chance-level (25% for set size 4, and 12.5% for set size 8). Shaded areas indicate standard errors. Colored dots indicate above chance-level decoding performance (*p* < .05, one-sided t test). b&d). Latency of decoding accuracies estimated via the jackknife-based procedure. *** indicates p < .001.

We applied MVPA to track target and singleton distractor representations across time. Both singleton distractor and target locations showed successful decoding (Fig 1). Target location representations emerged around 200 ms and persisted for most of the search period. Interestingly, the decoding of singleton distractor locations revealed an earlier initial peak, spanning from 100 ms to 200 ms. This initial peak was followed by a subsequent, more robust peak occurring between 200 ms and 400 ms for both set size 4, and set size 8. The decoding accuracies of singleton distractor locations quickly dropped off following the second peak whereas that for target locations persisted. The estimated latency for singleton distractor representations was faster than that for target representations in both singleton-present trials [experiment 1: *t*(19) = −5.17, *p* < 0.001, *d* = 1.58, *BF10* = 477.01; experiment 2: *t*(19) = −259.0, *p* < 0.001, *d* = 81.90, *BF10* = 1. 2 × 1031] and singleton-absent trials [experiment 1: *t*(19) = −9.92, *p* < 0.001, *d* = 3.15, *BF10* = 2. 0 × 10^6^; experiment 2: *t*(19) = −46.15, *p* < 0.001, *d* = 14.59, *BF10* == 4. 3 × 10^17^]. Interestingly, the latency for target representations in singleton-present trials was faster than in singleton-absent trials [experiment 1: *t*(19) = −11.86, *p* < 0.001, *d* = 3.87, *BF10* =3. 1 × 10^7^; experiment 2: *t*(19) = −12.11, *p* < 0.001, *d* = 3.79, *BF10* = 4. 4 × 10^7^].

**Fig. 2.**
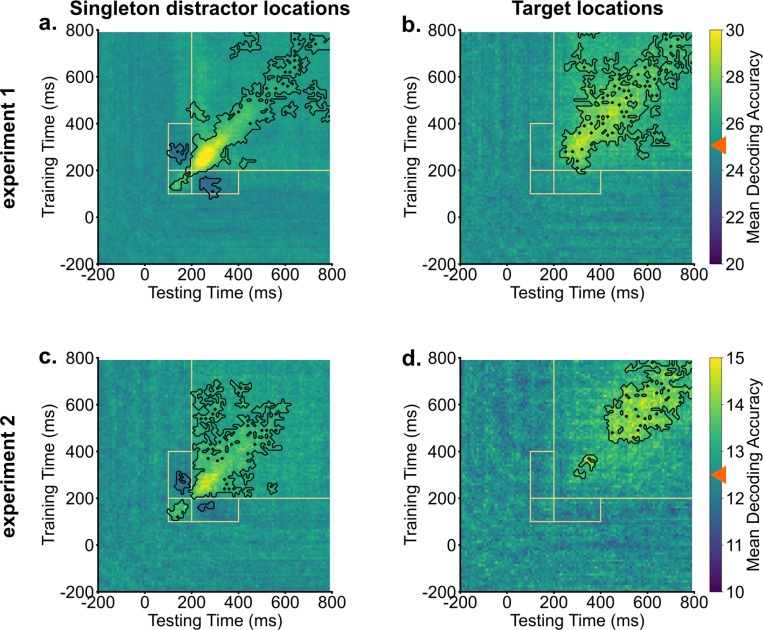
Singleton distractor representations showed inverted transformations, whereas target representations remained stable. Across experiments, generalization analyses of singleton distractor representations (a & c) revealed two clusters. Training decoders on activities from 100 ms to 200 ms resulted in below chance-level decoding evidence when tested on activities from 200 ms to 400 ms, and vice versa. Target representations demonstrated stability and could be generalized to neighboring time points (b & d). Orange boxes indicate key generalization time windows. Black contours indicate significant areas (*p* < .05). Bright colors indicate above chance-level decoding performance (denoted by orange triangles), and dark colors indicate below chance-level decoding performance.

The identification of two early peaks in singleton distractor decodings suggests potential shifts in representation during the search process. We conducted temporal generalization analyses to further examine these potential changes. Our methodology involved training decoders at one time point and then testing the decoder across all time points, repeating this procedure for each time point. As shown in Fig 2, target representations were stable over time. Trained decoders were able to generalize to neighboring time points. In contrast, the temporal generalization analysis of singleton distractor representations showed two discernible clusters. The initial cluster extended approximately from 100 ms to 200 ms, while the subsequent cluster emerged at around 200 ms. Crucially, these two clusters showed an inverted relationship. Decoders trained on activity from 100 ms to 200 ms had below-chance decoding performance when tested on data from 200 ms to 400 ms and vice versa. This negative generalizability performance suggests an inversion of representations for singleton distractors between the early (100 – 200 ms) and the later (200 – 400 ms) time windows. It also provides preliminary evidence for the signal transformation hypothesis that neural codings of singleton distractors were inverted during the search. However, it is unclear yet how this inversion directly supported attentional suppression.

### Singleton distractor representations facilitated visual search.

**Fig. 3.**
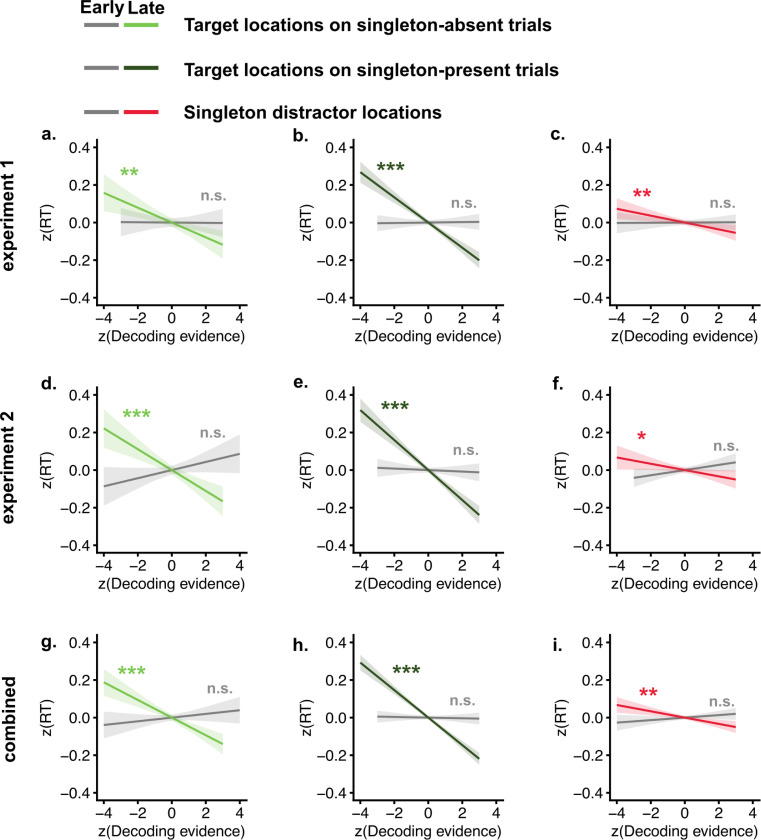
Target and singleton distractor representations predict faster reaction times. a–c) Target representations in singleton-absent, singleton-present, and singleton distractor representations predicted reaction time in experiment 1. Gray indicates the early window (100 – 200 ms); light green, dark green, and red indicate the late window (200 – 400 ms). d–f) Linear model results for experiment 2. g–i) Linear model results combining experiments 1 and 2. Shaded areas represent confidence intervals. n.s indicates non significant. * indicates p<.05, ** indicates p < .01, *** indicates p < .001.

We linked trial-by-trial decoding evidence for target and singleton distractor locations to reaction time to examine their functional roles. The relationship between early (100 – 200 ms) and late (200 – 400 ms) singleton distractor representations and reaction time is particularly informative for distinguishing between theoretical accounts of distractor processing. Under the reactive suppression framework, attentional suppression occurs after attention has been captured by the singleton; thus, early distractor representations could reflect attentional capture, whereas later representations would reflect reactive suppression. In contrast, the signal suppression hypothesis proposes that early distractor representations reflect sensory salience, while later representations reflect active suppression. Our linear modeling results (Fig 3) showed that only late target representations negatively predicted reaction times in both singleton-absent (exp1: *β* = −0.04, CI = [−0.06, −0.02], *p* = .001; exp2: *β* = −0.06, CI = [−0.08, −0.03], *p* < .001; combined: *β* = −0.05, CI = [−0.06, −0.03], *p* < .001) and singleton-present trials (exp1: *β* = −0.07, CI = [−0.08, −0.05], *p* < .001; exp2: *β* = −0.08, CI = [−0.10, −0.06], *p* < .001; combined: *β* = −0.07, CI = [−0.08, −0.06], *p* < .001), whereas early target representations did not ( *ps* > .05). Critically, early singleton distractor representations did not predict reaction times (exp1: *β* = 0.00, CI = [−0.01, 0.01], *p* = .942; exp2: *β* = 0.01, CI = [−0.00, 0.03], *p* = .083; combined: *β* = 0.01, CI = [−0.00, 0.02], *p* = .199), whereas late distractor representations negatively predicted reaction times (exp1: *β* = −0.02, CI = [−0.03, 0.00], *p* = .008; exp2: *β* = −0.02, CI = [−0.03, −0.00], *p* = .035; combined: *β* = −0.02, CI = [−0.03, −0.01], *p* = .001). Together, these findings indicate that both target and singleton-distractor representations in the late window are associated with faster reaction times and thus enhanced behavioral performance.

### Singleton distractor locations were suppressed in the spatial priority map.

**Fig. 4.**
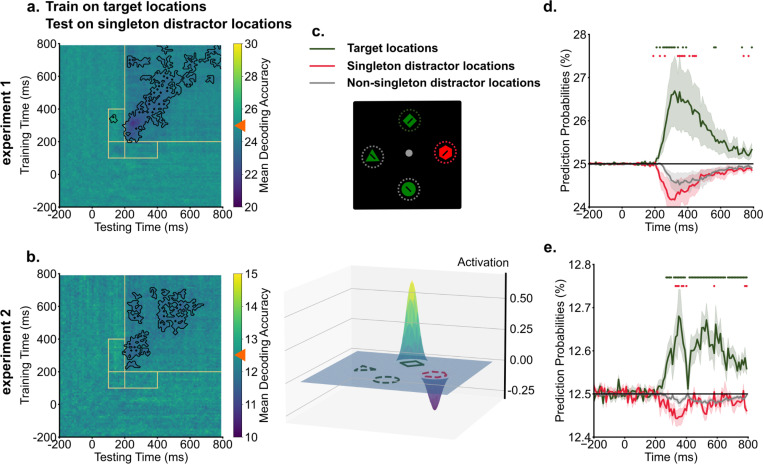
Singleton distractor locations were suppressed in the spatial priority map. a & b) Across experiments, training decoders on target locations from singleton-absent trials demonstrated below chance-level decoding performance (denoted by orange triangles) for singleton distractor locations, indicating the suppression of singleton distractor locations. Dark colors indicate below chance level decoding performance for singleton locations. Black contours indicate significant areas (*p* < .05). c) An example (250 ms in experiment 1) illustrates how we computed activation scores from decoding prediction probabilities. Prediction probabilities of target locations (green) and singleton distractor locations (red) were baselined to the averaged prediction probabilities of non-singleton distractor locations (gray). d & e) The time course of prediction probabilities for target locations and singleton distractor locations were plotted against the baseline (other locations). From 200 ms into the search, target locations showed increased prediction probabilities, whereas singleton distractor locations showed decreased prediction probabilities compared to the baseline. Shaded areas indicate standard errors. Colored dots indicate significant differences in prediction probabilities between target, singleton distractor locations to non-singleton distractor locations (*p* < .05).

To investigate how the inversion of singleton representations supported attentional suppression and facilitated visual search, we performed cross-condition generalization analyses. In this procedure, decoders were trained on target representations in singleton-absent trials, where top-down enhancements served as the primary guiding sources to boost target locations in the priority map. Subsequently, these trained decoders were tested on singleton-present trials to reveal the priority maps in instances where both target enhancement and singleton suppression might influence spatial priorities. The cross-condition generalization analyses revealed that singleton distractor locations were suppressed in the spatial priority map, resulting in below chance-level decoding performance (Fig 4.a & b). This suppression of singleton distractor locations started around 200 ms and persisted. Intriguingly, no generalization was observed between target representations and singleton distractor locations during the early time window from 100 ms to 200 ms, corresponding to the initial cluster of singleton distractor representation observed in temporal generalization analyses.

Below-chance decoding performance could reflect suppression of singleton distractor locations. Alternatively, it might be driven by a strong enhancement of target locations, which would reduce decoding performance for all other locations. To rule out the alternative possibility, we compared the spatial priority of singleton distractors against a baseline of non-singleton distractors in the search array. We derived prediction probabilities from trained decoders for each location in the search array and compared them across target locations, singleton distractor locations, and non-singleton distractor locations. Activation scores for target and singleton distractor locations were computed as the difference in prediction probabilities compared to the baseline (non-singleton distractor locations, for one example time point, see Fig 4.c). For between 200 ms and 400 ms into the search, we found enhancement of target locations compared to the baseline [experiment 1: *t*(19) = 2.14, *p* = 0.045, *d* = 0.81, *BF10* = 1.51; experiment 2: *t*(19) = 2.93, *p* = 0.009, *d* = 1.02, *BF10* = 5.78]. Critically, suppression of singleton distractor locations compared to the baseline was also observed across the experiment [experiment 1: *t*(19) = −2.11, *p* = 0.048, *d* = 0.32, *BF10* = 1.42; experiment 2: *t*(19) = −2.62, *p* = 0.017, *d* = 0.68, *BF10* = 3.30]. As shown in Fig 4.d & e, prediction probabilities of target locations displayed enhancement relative to the baseline from 200 ms onwards. In contrast, prediction probabilities of singleton distractor locations were suppressed below the baseline.

### Singleton distractor and target locations were coded in a shared neural space but in a reversed manner.

Cross-condition generalization analyses suggested that singleton distractors were suppressed in the neural space representing target locations. We hypothesized that this suppression might be driven by coding distractor information in an inverted format relative to target information. Such inverted coding of target and distractor information could facilitate the integration of top-down enhancement and suppression in computing spatial priority (Awh et al., 2012; Fecteau & Munoz, 2006; Itti & Koch, 2000; Luck et al., 2021). To directly test this hypothesis, we conducted neural correlation analyses to examine the relationship between target and singleton distractor representations.

**Fig. 5.**
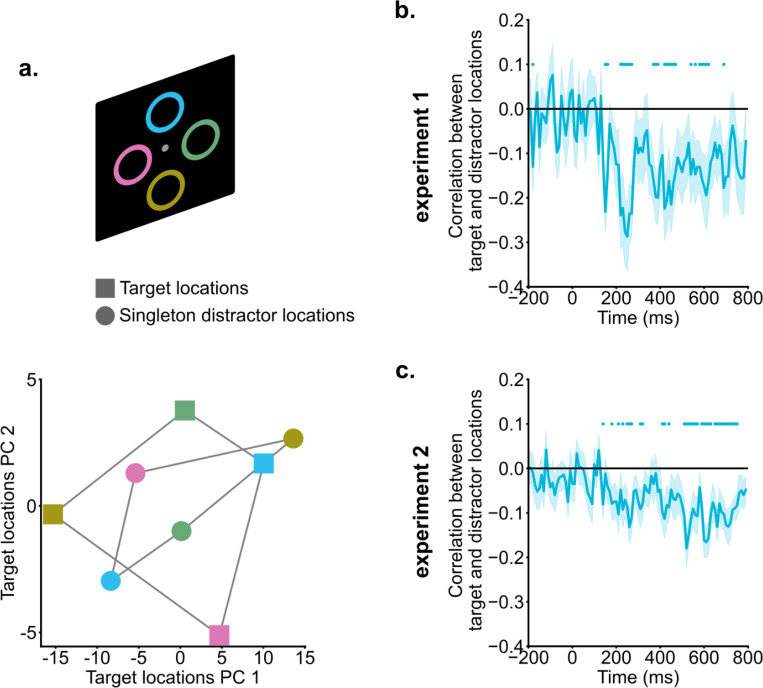
Singleton distractor suppression and target enhancement relied on inverted neural coding. a) Singleton distractor and target representations of an example subject were projected onto a 2D target representational space for illustration purposes. Different colors indicate different locations. Singleton distractor and target representations exhibited an inverted pattern. b & c) Correlations between singleton distractor and target representations. Starting from around 200 ms, singleton distractor and target representations showed negative correlations. Shaded areas indicate standard errors. Colored dots indicate significant correlations compared to zero (*p* < .05).

In Fig 5.a, representations of both targets and singletons were shown for an example subject from 200 ms to 400 ms into the search, projected in a 2D subspace for easier visualization. This subspace was constructed using the first two principal components (a total of 99% variance explained) of the neural representations of target locations. The location information demonstrated clear organization; the responses to four locations were well separated, and the distance between neural representations corresponded closely to the physical distance between locations (i.e., neighboring locations had neighboring representations). Interestingly, singleton distractor and target representations showed a reversed pattern. For instance, the top location (blue square) in the target representational space was projected to the top-right corner, whereas the same location in the singleton distractor representational space was projected to the bottom-left region (blue circle).

Formal correlation analyses were performed to link target representations and singleton distractor representations in the raw signal space (dimension = channel). For between 200 ms and 400 ms into the search, target and singleton distractor representations showed reliable negative correlations [experiment 1: *t*(19) = −3.22, *p* = 0.005, *d* = 0.72, *BF10* = 9.99; experiment 2: *t*(19) = −3.05, *p* = 0.006, *d* = 0.68, *BF10* = 7.29]. As shown in Fig 5.b &c, negative correlation emerged around 200 ms and persisted into the later search period, suggesting that singleton distractor representations and target representations were coded with an inverted representational geometry.

## Discussion

In recent years, attention research has shifted from a primary focus on target enhancement to an increasing interest in distractor suppression (Geng et al., 2019; Luck et al., 2021; Theeuwes, 2025; Wöstmann et al., 2022). While growing evidence shows that attention can be directed away from cued or learned distractors (Arita et al., 2012; Chang & Egeth, 2019; Gaspelin et al., 2015, 2017; Sawaki & Luck, 2010; Vatterott & Vecera, 2012; Won et al., 2019; Zhang et al., 2020, 2022; Zhang & Carlisle, 2023), it remains unclear how such suppression unfolds over time, particularly how to-be-suppressed information is represented in the brain. The primary goal of this study was to test a novel mechanism of signal transformation underlying singleton distractor suppression in visual search. Using MVPA applied to EEG data, we compared the temporal trajectories of target and singleton distractor representations. We found that singleton distractor representations were reliably represented earlier than targets, consistent with the idea that salient distractors elicit rapid bottom-up signals before top-down target enhancement emerges (Jonides & Yantis, 1988; Theeuwes, 2010). Increasing set size from four to eight items delayed target representations but did not affect singleton distractor representations, in line with the finding that crowding weakens target signals (Reddy & VanRullen, 2007), whereas salience-based singleton signals remain stable. Interestingly, the latency for target representations in singleton-present trials was faster than in singleton-absent trials. This pattern could suggest that the presence of a singleton distractor facilitates the emergence of target representations. However, it could also reflect a limitation of using spatial decoders in arrays where target and singleton locations are mutually exclusive, as we discuss in detail in the following section.

We identified two distinct peaks in decoding evidence for singleton distractor locations. To examine potential changes in representations associated with the two peaks, we conducted temporal generalization analyses. Target representations remained stable across time, forming a consistent cluster from around 200 ms to subsequent times in the search. In contrast, singleton distractor representations showed two separate clusters: an early one from 100 ms to 200 ms, followed by a larger cluster from 200 ms to later search periods. Importantly, these two clusters of singleton distractor representations showed negative generalizability, indicating an inverted transformation of coding formats around 200 ms in the search. We next examined how inversions of singleton distractor representations influence spatial priority computations. Decoding models were trained on target locations from singleton-absent trials to capture spatial priorities primarily driven by top-down target enhancement. These trained decoders were then applied to singleton-present trials to reveal the spatial priority structure in the presence of a singleton distractor. This cross-condition generalization analysis showed below-chance decoding performance at singleton distractor locations. Below-chance decoding could arise either from enhanced target representations, which would push prediction probabilities for all non-target locations below chance, or from suppression specifically targeting the singleton distractor location. To distinguish these possibilities, we computed prediction probabilities for each location in the search array and used the average of the non-singleton distractor locations (two in experiment 1 and six in experiment 2) as a baseline. Relative to this baseline, target locations showed above-baseline enhancement beginning around 200 ms. Critically, singleton distractor locations showed below-baseline suppression around the same time, providing converging evidence that singleton distractor representations were actively suppressed within the spatial priority map.

A key limitation of using spatial decoders for probing spatial priority is that target and singleton distractor locations are inherently anticorrelated. Negative decoding evidence may therefore reflect either suppression of a specific location or enhancement of the target location. Although we attempted to address this issue by comparing singleton distractor locations with non-singleton distractor locations, and found that singleton distractor locations were indeed lower than these controls, this inherent limitation cannot be fully resolved with such comparisons. Future studies should directly assess suppression using alternative approaches, such as feature decoders or frequency tagging (Forschack et al., 2017; van Moorselaar et al., 2020), particularly in designs where targets and singleton distractors vary along distinct feature dimensions.

Finally, we turned to the pivotal question of how singleton distractor representations were suppressed. We hypothesized that suppression is driven by coding singleton distractor representations in an inverted format relative to target representations. Such inverted coding could facilitate the downstream readout and integration of both target enhancement and distractor suppression. To test this idea, we conducted correlations between singleton distractor and target representations. The analysis revealed robust negative correlations starting around 200 ms into the search, indicating that target locations and singleton distractor locations were indeed inversely coded within a shared neural space. This shared neural space likely plays a critical role in the computation of spatial priorities, where target signals and singleton distractor signals generate opposing enhancement and suppression effects. While these findings offer valuable insights, further evidence is required to rigorously test the relationship between the target and singleton distractor subspaces and the hypothesized shared spatial priority map.

While accumulated evidence from various modalities, including behavioral, eye-tracking, electrophysiological, EEG, and fMRI data supports the successful suppression of salient color singleton distractors, the neural mechanisms underlying this phenomenon remain less clear (Chang & Egeth, 2019; Cosman et al., 2018; Gaspelin et al., 2015, 2017; Sawaki et al., 2012; Sawaki & Luck, 2010; Stilwell et al., 2022; Vatterott & Vecera, 2012). Previous electrophysiological studies in NHPs have demonstrated that neuronal activity elicited by salient singleton distractors can be suppressed relative to the activity elicited by non-singleton distractors in areas of FEF, LIP, and V4 (Cosman et al., 2018; Ipata et al., 2006; Klink et al., 2023). Additionally, human fMRI studies have indicated that representations of singleton distractors are suppressed across multiple early visual regions and parietal regions involved in computing spatial priorities (Adam & Serences, 2021; Won et al., 2019). These findings align with the broader idea that suppression may involve the attenuation of singleton distractor signals, particularly within spatial priority maps that guide attention away from distractor locations. However, recent research points to an alternative possibility of signal transformation rather than attenuation. First, neural responses to singleton distractors appear to be highly heterogeneous. Klink et al. (2023) reported a transient enhancement followed by later suppression of V4 activity in response to singleton distractors. A follow-up study by Sapountzis et al. (2025) identified three subpopulations of neurons in FEF and LIP, those showing persistent enhancement, persistent suppression, or an early enhancement followed by later suppression, indicating that singleton distractor processing cannot be fully explained by uniform signal attenuation. Intriguingly, multivariate decoding analyses have shown that singleton distractor representations persist rather than disappear, further suggesting that these representations may play a functional role in supporting the behavioral effects of attentional suppression.

**Fig. 6.**
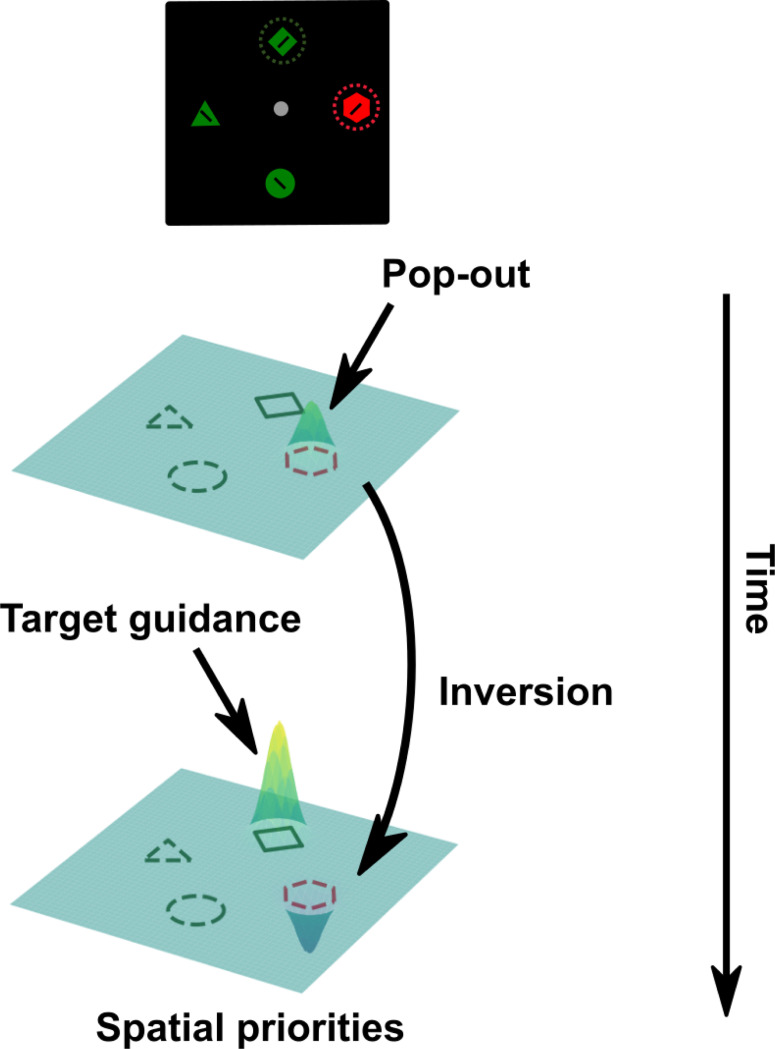
Proposed singleton suppression mechanism within the priority map framework. The process initiates with the registration of singleton distractor information. Subsequently, distractor representations undergo inversion, around the same time as the emergence of target representations. Both distractor and target representations are coded within a shared neural space but in an inverted manner. The representations within this neural space are then accessed to compute the ultimate spatial priorities that guide visual attention.

The present study extends prior work by directly examining representational transformations in singleton distractor processing. We identified a rapid inversion of singleton distractor representations early in the visual search, leading to those representations being coded in an inverted format relative to target representations. These findings provide compelling evidence for a representational transformation mechanism, rather than representational attenuation, as a basis for singleton suppression. Based on this evidence, we propose that attentional suppression begins with registering sensory inputs from the visual environment, with a singleton distractor being quickly encoded due to its saliency. The early singleton distractor representations we observed between 100 – 200 ms likely reflects this initial registration. Importantly, this early saliency signal does not necessarily lead to attentional capture. Instead, suppression appears to involve transforming this initial signal into a format that downregulates the singleton distractor’s priority within a spatial priority map (Fig. 6). The later singleton distractor representations are inverted relative to target representations and they predict faster reaction times, which together supports the idea that initial saliency signals are transformed into representations that facilitate distractor suppression. The timing of this transformation coincides with the emergence of target representations, suggesting that it may be driven by feedback from top-down control mechanisms that support target guidance, rather than by a purely feedforward attenuation of saliency. Future neuroimaging studies will be essential for characterizing the temporal and spatial dynamics of these processes and for delineating the similarities and differences between target guidance and distractor suppression.

Major hypotheses regarding singleton suppression, including the signal suppression hypothesis (Gaspelin & Luck, 2018b; Sawaki & Luck, 2010) and the rapid disengagement hypothesis (Theeuwes, 2010), both suggest a transformation from an initial priority signal to eventual suppression signals for singletons, but differ in the hypothesized timing of these transitions. The signal suppression hypothesis proposes that inhibition of the salient singleton happens prior to the initial shift of visual attention (proactive suppression), whereas the rapid disengagement hypothesis suggests that suppression occurs only after singletons have captured attention (reactive suppression). Our temporal generalization analysis identified representational transformation of singleton distractors around 200 ms after the onset of the search array which persisted throughout the remainder of the trial.

Importantly, the initial singleton distractor representations observed from 100 – 200 ms were coded in a reversed format relative to later representations (200 – 400 ms), and in a format distinct from target representations at any stage of the search. At face value, this transformation appears consistent with the reactive suppression account (Theeuwes, 2010; Theeuwes, 2023), which posits that attention is first captured by the singleton and then actively suppressed. However, under the signal suppression hypothesis, these early singleton signals may instead reflect the sensory registration of salient singleton distractors, and those signals might not directly affect attention unless they are subsequently incorporated into the priority map (Gaspelin & Luck, 2018b). To further clarify their functional role, we linked decoding evidence of singleton distractor locations to reaction times. Early singleton distractor representations did not predict reaction times, whereas later singleton distractor representations reliably predicted faster responses. This pattern indicates that early singleton signals are unlikely to reflect attention capture; instead, they likely index the initial registration of distractor information. In contrast, late singleton distractor representations were associated with faster target responses, and thus these neural signals likely reflect distraction suppression. To fully rule out the possibility that early singleton distractor representations reflect attention capture, future research will need to determine whether the guiding signals associated with top-down goals and bottom-up salience operate independently or share overlapping representational formats.

A limitation of the study is that the dataset does not include eye-tracking data, making it impossible to directly test whether the observed transformation of singleton distractor representations is a reactive consequence of initial attentional capture. However, this interpretation is unlikely for two reasons. First, attentional capture by singleton distractors is rarely observed in studies using highly similar paradigms, with reported capture rates typically below 10% (Gaspelin et al., 2017; Stilwell et al., 2023; Adam et al., 2023). Across these studies, initial saccades are reliably biased away from singleton-distractor locations, including for the fastest quartile of saccades (Gaspelin et al., 2017; Stilwell et al., 2023). Second, even in the small proportion of cases where saccades do land on the singleton distractor, their latencies are approximately 200 ms (e.g., 215 ms, 194 ms, and 185 ms across three experiments in Gaspelin et al., 2017; 218 ms, 210 ms, 204 ms, and 200 ms across four experiments in Adam et al., 2023; see also Adam et al., 2021; Stilwell et al., 2023). This timing coincides with the onset of the representational transformation observed in the current study. Thus, even if attentional capture occurs, it happens around the same time as the observed transformation. Taken together, these findings suggest that the observed signal transformation is more consistent with a proactive mechanism and is unlikely to reflect a reactive response to attentional capture by singleton distractors. It is also worth noting that the distinction between these two accounts may not be as substantial as traditionally assumed. Recent modeling work has shown that a single underlying mechanism, preventing the early singleton distractor signal from triggering a saccade, can account for results from paradigms demonstrating both attentional suppression and attentional capture (Zhang et al., 2025). This framework suggests that these ostensibly distinct accounts of suppression may, in fact, arise from a shared underlying mechanism.

Our interpretations of the two distinct stages of singleton distractor representations parallel the interpretations of two temporally dissociable ERP components associated with singleton distractor processing. An early positive-going deflection, the Ppc component (approximately 80 – 150 ms), is reliably observed in arrays containing lateralized singleton items, regardless of whether the singleton is the target or a distractor. This early component has been interpreted as reflecting the initial saliency signal or low-level sensory imbalance across hemifields (Barras & Kerzel, 2016, 2017; Fortier-Gauthier et al., 2012; Leblanc et al., 2008; Oxner et al., 2024; van Moorselaar et al., 2023, 2025). A later positive-going component, the Pd (with a broad latency range across studies, roughly 100 – 500 ms, and typically 100 – 275 ms for the early Pd) has been associated with the inhibitory processes involved in handling singleton distractors (Hickey et al., 2009; Gaspar & McDonald, 2014; Sawaki & Luck, 2010; for a recent review, see Gaspelin et al., 2023). Although these ERP components can be difficult to disentangle due to overlapping electrodes and partially overlapping time courses, multivariate decoding approaches can avoid this issue to some extent by capturing activity patterns in a high dimensional representational space. Our findings largely align with these ERP components and extend current understanding by providing evidence for representational shifts that may bridge the two proposed stages of singleton distractor processing.

The efficiency of visual search relies on various guiding signals, including information about relevant goals (goal-driven), previous search experience (experience-driven), and the salience determined by local image statistics (stimulus-driven) (Awh et al., 2012; Kim & Anderson, 2019; Luck et al., 2021; Narhi-Martinez et al., 2023). These diverse guiding signals are integrated somehow into topographically organized priority maps, which direct visual attention based on the relative strength of priority signals among candidate locations (Awh et al., 2012; Fecteau & Munoz, 2006; Itti & Koch, 2000; Luck et al., 2021). While this framework of priority maps is valuable for studying multiple sources of guiding signals, only a limited number of neuroimaging studies have explored how spatial priorities are computed and utilized for guiding attentional suppression (Adam & Serences, 2021; Serences & Yantis, 2007; Sprague et al., 2018; Sprague & Serences, 2013). Our study estimated changes in spatial priorities using cross-condition generalization analyses and found evidence of suppression at singleton distractor locations. However, this approach relies on key assumptions that a single priority map integrates multiple sources of guidance (Wolfe, 2021; Gaspelin et al., 2025; Liesefeld et al., 2024; Theeuwes et al., 2022), and that the neural implementation of the priority map used in singleton-absent trials is shared with that used in singleton-present trials. These assumptions remain open questions, given ongoing debates about whether multiple, functionally distinct priority maps exist across the cortex (Bisley & Mirpour, 2019; Todd & Manaligod, 2018). Our findings suggest that a common priority map can support both attentional enhancement and distractor suppression. Yet it is also plausible that multiple priority maps are instantiated in different brain regions, or in different representational subspaces within the same regions, allowing target enhancement and distractor suppression to operate in parallel. For example, when target and distractor features become predictable through learning, target enhancement and distractor suppression have been shown to rely on distinct neural mechanisms (van Moorselaar et al., 2019, 2020). A related limitation of our approach is that we cannot disentangle the priority map specifically encoding top-down guidance signals from the overall priority map, because in singleton-absent trials, top-down guidance dominates the priority structure. Disentangling the final spatial priority map from the subspaces that encode different guiding signals remains a challenge. Future research is needed to clarify how these distinct sources are represented and transformed to compute spatial priorities.

In conclusion, our findings elucidate a novel mechanism of representation inversion underlying singleton distractor suppression in visual searches. We observed a rapid inversion of distractor representations early in the search process. These inverted representations are coded in a shared neural space with target representations but exhibit an inverse relationship, leading to the suppression of singleton locations in the estimates of spatial priorities to guide behavior.

## Significance Statement

A primary goal of attention is to select relevant information while ignoring irrelevant input from the external environment. Although the mechanisms of attentional enhancement have been extensively studied, the mechanisms underlying attentional suppression remain less well understood. While prior work has made important progress in identifying when attentional suppression is engaged, the neural mechanisms that implement suppression are still unclear. Here, we show that neural representations of salient singleton distractors undergo a rapid inversion approximately 200 ms after search onset. These inverted representations can subsequently be read out as suppression signals during the computation of spatial priorities. Together, our findings suggest that transforming initial pop-out distractor signals into an inverted representational format might drive rapid attentional suppression and support goal-directed visual search.
